# Prevalence of *Helicobacter pylori* in asymptomatic patients at surgical outpatient department: Harare hospitals

**DOI:** 10.1016/j.amsu.2018.09.040

**Published:** 2018-09-28

**Authors:** Simbarashe Gift Mungazi, Onesai Blessing Chihaka, Godfrey I. Muguti

**Affiliations:** Department of Surgery, College of Health Sciences, University of Zimbabwe, Box A167, Avondale, 263, Harare, Zimbabwe

**Keywords:** *Helicobacter pylori*, Asymptomatic, Prevalence

## Abstract

**Background:**

*Helicobacter pylori* infection is present in more than 50% of the world's population. The estimated life time risk of peptic ulcer disease is 20 percent and of gastric cancer is 1–2 percent.

**Materials and methods:**

A cross sectional study was done at two Central hospitals in Harare, Zimbabwe, with the objective being to estimate the prevalence of *Helicobacter pylori* infection in asymptomatic individuals. Other objectives were to determine the association of the *Helicobacter pylori* infection with potential risk factors.

Four hundred and fifty patients visiting the outpatient surgical clinics for other complaints other than upper gastrointestinal symptoms were recruited in the study. Drops of whole blood were obtained by fingertip puncture from each patient. The Onsite *H. pylori* Combo Rapid Test was used to confirm the presence or absence of antibodies against *Helicobacter pylori*. A questionnaire was used to record the sociodemographics of the participants.

**Results:**

Three hundred patients, 186 males (62%) and 114 females (38%) participated. The prevalence of *Helicobacter pylori* infection was 67.7 percent (203/300). The prevalence of H pylori infection was significantly correlated with increasing age (p = 0.012), sharing of a bed with siblings during childhood (p = 0.013) and the mode of sanitation methods (p = 0.023). There was no association found between H pylori infection and other risk factors such as; gender, level of education, employment status or number of rooms in a house.

**Conclusion:**

*H. pylori* infection prevalence was significantly associated with increasing age, sharing of a bed with siblings during childhood and the mode of sanitation used. Clinicians and the public have to be aware of the important role of H pylori in upper gastrointestinal disease. Use of better sanitation methods, appropriate hygiene, avoidance of over-crowding amongst other measures should be encouraged as a means to reduce the acquisition and transmission of H pylori.

## Highlights

• Prevalence of *H. pylori* infection is 67.7%.
• The results are from Zimbabwe.
• It is associated with increasing age and the mode of sanitation.
• It is associated with sharing of a bed with siblings during childhood.

## 1 Introduction

*Helicobacter pylori* (*H. pylori*) is a spiral shaped, gram negative, microaerophilic bacterium that persistently colonizes the gastric mucosa of humans. The estimated lifetime risk of peptic ulcer disease is 20 percent and gastric cancer is 1–2 percent with H pylori infection [1]. The prevalence of H pylori infection is as low as 14 percent in developed countries and as high as 92 percent in under developed countries [[2], [3], [4]]. The spread and acquisition of *H. pylori* has generally been linked to a number of factors including crowding/density, poor sanitization methods, social factors (such as smoking), waterborne exposure, occupational exposure and poor hygienic practices [5]. Epidemiological knowledge of *H. pylori* infection in early studies emphasized on *H. pylori* infection in symptomatic patients presenting for endoscopy and hence little information is known about the frequency of H pylori in the general population [6]. This study enhances the knowledge of the important role of H pylori in upper gastrointestinal disease worldwide.

The specific objectives of this study were to:

1. Estimate the prevalence of *Helicobacter pylori* in asymptomatic individuals and,
2. Determine the association of the *Helicobacter pylori* with potential risk factors such as age, gender and the sociodemographic status (level of education, number of rooms and family member living in/with, source of drinking water, sharing of a bed and animal ownership).

## 2 Materials and methods

A cross-sectional study was done. Sample size calculated using the Dobson's formula was 185. Between July 2014 to November 2014 four hundred and fifty patients were approached for the study.

Patients visiting the outpatient surgical clinics at two central, public and teaching hospitals for other complaints other than upper gastrointestinal symptoms were recruited in the study. The targeted surgical clinics were the general surgery, orthopaedic, urology, cardiothoracic, neurosurgery and paediatric surgery clinics Eligibility criteria was all individuals who did not have upper gastrointestinal symptoms presenting to the surgical outpatients' clinics. Patients were excluded if; a) they had upper gastrointestinal tract symptoms such as epigastric pain, indigestion and nausea/vomiting, b) they had a history of peptic ulcer disease or any use of antacids regularly, c) they took antibiotics for the past 6–8 weeks, d) they were below the age of 1 year and e) if patients refused to be included in the study. The patients were recruited in the order of who was first in the outpatients' queue.

Drops of whole blood were obtained by fingertip puncture from each patient. The commercial sandwich lateral flow chromatography kit (Onsite H. Pylori Combo Rapid Test, CTK Biotech) was then used to detect the presence of antibodies; immunoglobulin (Ig) G, Ig M or Ig A to confirm presence or absence of *Helicobacter pylori*. The Onsite *H. pylori* Combo Rapid Test has a relative sensitivity of 86.7 percent and relative specificity of 91 percent. A questionnaire was used to record the age, sex and socioeconomic status of the participants.

All data from data collection sheet was entered into a computer using Epidemiological Information -programme software and was analyzed using Statistical Package for Social Scientist (SPSS) version 16. Descriptive statistics were used to report measures of central tendencies for quantitative variables. Student's t-test for independent groups was used to test and also check relationships on continuous variables. T-test were two tailed. Categorical variables were expressed as percentages and frequencies, and compared using the Chi-square analysis. Graphs were used to present categorical variables in pictorial view. Statistical analysis was carried out and P-value of 0.05 was considered significant at 95% confidence interval.

## 3 Results

**Fig. 1:**
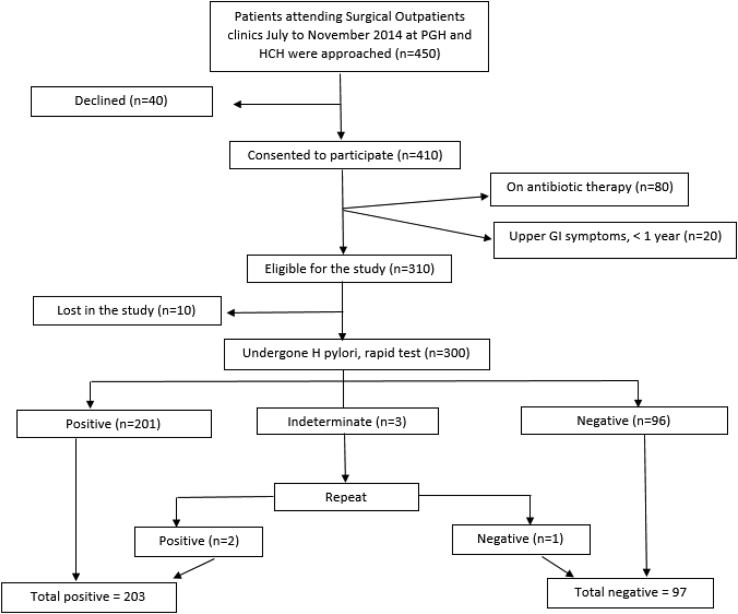
Flow chart.

The flow chart (Fig. 1) below shows the results of the recruitment of patients. Three hundred individuals were recruited for the study. Most of the patients (n = 80) excluded from the study had been on antibiotics. Ten patients who were eligible and had agreed, did not turn up for the study after they been attended for their primary presenting complaints.

### 3.1 Demographic characteristics of the study population

The age range was 1 year–94 years. The mean age was 35.6 and standard deviation ± 25.3. There were a total 186 males (62%) and 114 females (38%) that participated in the study.

### 3.2 Prevalence of *H. pylori* infection

The study showed that of the 300 patients, 67.7% (n = 203), were positive for *H. pylori* infection whilst 32.3% (n = 97), were negative.

### 3.3 *H. pylori* prevalence by sex and age group

**Fig. 2:**
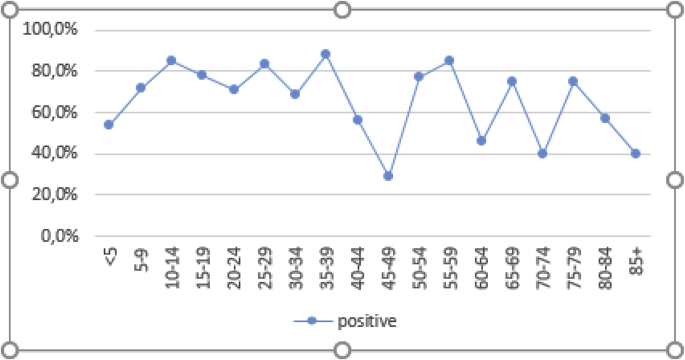
*H. pylori* by age.

The prevalence of *H. pylori* was higher in males (71.0%) as compared to females (62.3%). However, the differences were not statistically significant (p = 0.076). There was an increase in H pylori infection prevalence with an increase in age. This was statistically significant (p = 0.012) (Fig. 2).

### 3.4 Association of *H. pylori* with potential risk factors

There was no association between *H. pylori* infection and the level of education, occupation or the type of residence (Table 1).

**Table 1:** Association of *H. pylori* infection with sociodemographic characteristics.

Variables		Number	*H. pylori* positive + (%)	P value
Level of education	Illiterate	9	4	0.105
	Primary School	94	35	
	Secondary School	155	42	
	High School	13	3	
	College/University	19	10	
Occupation	Not employed	205	64	0.384
	Formally Employed	92	31	
	Housekeeping	12	2	0.509
	Blue collar	56	20	
	White collar	24	9	
	Self employed	43	12	
Residence	Rural	89	29	0.527
	Urban	211	68	
Housing tenure	Owned	198	65	0.452
	Rented	102	32	
Household population(persons/home)	1–3	58	25	0.08
	4–5	87	22	
	6+	155	50	
Rooms occupied	1–2	47	17	0.739
	3–4	99	28	
	5–6	72	25	
	7+	82	27	

### 3.5 Association between *H. pylori* and income

Fifty-one patients of the 300 were formally employed. There was no association between H pylori and income (p = 0.741).

### 3.6 Association between *H. pylori* with sanitation method and with living conditions

There was a high H pylori infection prevalence in patients who used the Blair toilet (78,6%) as a mode of sanitation compared to Flush toilet (65,4%) and pit latrine (57,7%) (p = 0.023).

*H. pylori* infection prevalence was significantly associated with sharing of a bed with a sibling during childhood (p = 0.013). There was no association between *H. pylori* infection and other living conditions. (Table 2).

**Table 2:** Association of *H. pylori* with living conditions.

Variables		Number	*H. pylori* + (%)	P value
Sharing of bed with parents during childhood	No	99	32	0.998
	Yes	201	65	
Sharing of the bed with siblings during childhood	No	77	44	0.013
	Yes	223	159	
Source of drinking water	Borehole	125	36	0.547
	City water	112	41	
	Well	62	20	
	River	1	0	
Sharing of sanitation methods	No	104	39	0.172
	Yes	195	58	
Animal ownership	No	163	59	0.119
	Yes	137	38	

## 4 Discussion

**Fig. 3:**
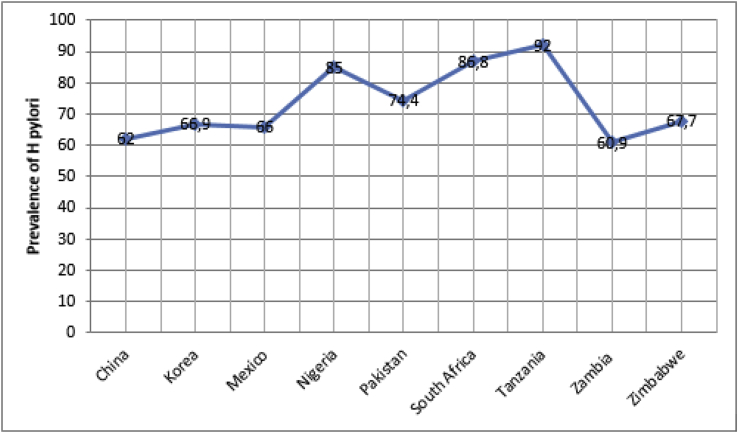
*H. pylori* according to country.

This study showed the prevalence of H pylori infection to be 67.7%, a high prevalence which is similar to other studies conducted in developing countries. A prevalence of 86.8% was found among a South African population [7], 60.9% was found in Zambian population [8] 90% in children and 85% in adults in Nigeria [9,10]. Other studies showed the same trend of H pylori infection rates being high; 92% in Tanzania [2], 66.9% among Korean population [11], while a prevalence of 74.4%, 66% and 62% was found among Pakistan [12], Mexican [13,14] and Chinese [15] population respectively see Fig. 3. Zimbabwe is still a developing country where poor methods of sanitation, overcrowding and low socioeconomic status contribute to the high prevalence of *H. pylori* as noted in this study. The study population consisted mostly of black Zimbabwean population.

In the current study more males (71%) were positive for H pylori infection compared to females (62.3%) although this was not statistically significant (p = 0.076). Reports by Moayyedi et al. [16] and Ndip et al. [17] showed a higher prevalence in males than in females. In both these studies the gender difference was statistically significant, p = 0.015 and p < 0.05 respectively. The explanation for the likely gender difference in H pylori prevalence is not clear but maybe due to young boys having poorer hygiene than girls [18].

The current study found that the prevalence increased with age from 53.5% in children <5 years to 88% in adults aged between 35 and 39 years. The increase in prevalence was statistically significant (p = 0.012). The study findings are similar to studies in South Africa [7]and Pakistan [12]. Early acquisition of H pylori infection was demonstrated in our study as in other studies, with H pylori infection prevalence noted to be 53.5% at <5 years and 71.4% at 5–9years. This is consistent with literature elsewhere [7,12]. In Nigeria, Holcombe et al. [9] noted that H pylori infection is common from an early age in the developing world, where most children are colonized by the age of 10 years. These high prevalence rates of H pylori infection in early childhood were also noted in Tanzania [2] and Cameroon [17]. As noted in this study infection rates are acquired in early childhood, research therefore should focus on the mode of transmission during early childhood. Children as well as adults should be included in intervention programmes designed to treat H pylori infection, especially in a population with a high risk of gastric cancer like China as noted by Jun-ling et al. [19].

The standard of education is a strong indicator of socioeconomic class [20]. In our study there was no association between H pylori infection and the level of education. Rasheed et al. [12] also found no association between H pylori infection and the level of education. This is not in agreement with the EUROGAST study group [20], Torres et al. [13]and Graham et al. [6] studies that showed a strong link between a high level of H pylori prevalence amongst the illiterate. The disproportionate sample size 155 out of 300 (51.7%) being educated up to secondary school compared to 9 out of 300 (3%) who did not attend any form of education could be a possible explanation for the findings in our study.

There was no association between H pylori prevalence with employment status, residency (urban vs rural).

Whilst the spread of the H pylori organism has been shown to be associated to water [5], our study did not find any association between H pylori infection and the source of drinking water (p = 0.547), a similar finding in the study by Rasheed et al. [12].

In the present study, there was a high H pylori infection prevalence in individuals who shared a bed with their siblings during childhood, 71.3% compared to those who did not 57.1% (p = 0.02). This is in keeping with studies done by Moayyedi [16]and Rothenbacher [21]. Interestingly the association between H pylori infection and those who shared a bed with their parents was not statistically significant, (p = 0.998), this was similar to findings in the study by Moayyedi et al. [16] (p = 0.65). The probable explanation being that since H pylori acquisition is mostly during childhood, then a high intra family spread would be more likely between siblings. In addition, poorer hygienic practices in childhood may play a role in the association of H pylori and sharing of a bed amongst siblings as compared to sharing a bed with adults.

H pylori infection was high with the use of a Blair toilet (78.6%) and the association of H pylori infection with the type toilet was statistically significant (p = 0.023). However, there was no significant association between H pylori infection with toilet sharing with other families (p = 0.119). The public have to be aware of the important role of H pylori in upper gastrointestinal disease. Use of better sanitation methods, appropriate hygiene, avoidance of over-crowding amongst other measures should be encouraged as a means to reduce the acquisition and transmission of *H. pylori*.

A previous study by Rasheed et al. [12] reported a significant association of H pylori infection with the presence of pets. This study did not find an association between the two. Although Rothenbacher et al. [21] reached the similar conclusion, he further showed by a bivariate analysis a strong association between H pylori infection status and keeping a cat. Interestingly, Graham et al. [6] showed that having pets was associated with a lower frequency of H pylori infection.

## 5 Conclusion

In this study, the prevalence of *H. pylori* infection in asymptomatic population was 67.7%, which is comparable with data from other studies. *H. pylori* infection prevalence increased with age. There is no sex predilection with regards to *H. pylori* infection. H pylori infection was high in individuals who shared a bed with their siblings during childhood and those who used Blair toilets as a mode of sanitation. In this study there was no association between H pylori infection and level of education, source of drinking water, number of living rooms and number of family members in a household. Clinicians and the public have to be aware of the important role of H pylori in upper gastrointestinal disease. Use of better sanitation methods, appropriate hygiene, avoidance of over-crowding amongst other measures should be encouraged as a means to reduce the acquisition and transmission of H pylori.

## 6 Limitations

The derivation of the associated risk factors via a questionnaire which would be subject to bias is a limitation of the study. Two diagnostic tests would have increased the diagnostic power of the study. Use of ELISA would have been superior to finger prick test. Community based study as opposed to hospital based will in future be considered in future studies.

## Ethical approval

Ethical approval was obtained from Joint Research Ethics Committee for the University of Zimbabwe, College of Health Sciences and Parirenyatwa Group of Hospitals (JREC), JREC Ref: 142/14 and the Harare Central Hospital Ethics Committee Reference: 060814/46.

## Sources of funding

There was no source of funding.

## Author contribution

Simbarashe Gift Mungazi: project design, data collection, subject research, analysis and interpretation of data, writing and consent Onesai B Chihaka: subject research, analysis and interpretation of data, writing, editing Godfrey I Muguti: project design, subject research, analysis and interpretation of data, writing, editing.

## Conflicts of interest

There is no conflicts of interest.

## Research registration number

Researchregistry UIN 2822.

## Guarantor

Simbarashe G Mungazi.

Godfrey I Muguti.

## Provenance and peer review

Not commissioned, externally peer reviewed.

## Appendix A Supplementary data

checklist checklist
