# Supplementary material for: Prevalence of Helicobacter pylori in asymptomatic patients at surgical outpatient department: Harare hospitals
Source: Ann Med Surg (Lond). 2018 Sep 28;35:153–7. doi: 10.1016/j.amsu.2018.09.040 (PMC6174827; doi:10.1016/j.amsu.2018.09.040)
Supplement: checklist [file mmc1.docx]

| The STROCSS Guideline | | |
| --- | --- | --- |
| Item no. | **Item description** | Page Number |
| 1 | Title. The words “cohort” and the area of focus should appear in the title (e.g. disease, exposure/intervention or outcome). Whether the study is retrospective or prospective should also be stated. | The study was a cross sectional study |
| 2a | Abstract - Introduction What is the background and scientific rationale for the research question. | 1  Helicobacter pylori infection is present in more than 50% of the world’s population. The estimated life time risk of peptic ulcer disease is 20 percent and of gastric cancer is 1-2 percent. Research question being what is the prevalence of H pylori in asymptomatic patients. |
| 2b | Abstract - Methods - Describe the study design (cohort design, retrospective or prospective, single or multi-centre, etc), what was done to each group, how, when was it done and by whom. | 1  **:** A cross sectional study was done at two Central hospitals in Harare, Zimbabwe with the objective being to estimate the prevalence of Helicobacter pylori infection in asymptomatic individuals. Other objectives were to determine the association of the Helicobacter pylori infection with potential risk factors.  Four hundred and fifty patients visiting the outpatient surgical clinics for other complaints other than upper gastrointestinal symptoms were recruited in the study. Drops of whole blood were obtained by fingertip puncture from each patient. The Onsite H. pylori Combo Rapid Test was used to confirm the presence or absence of antibodies against Helicobacter pylori. A questionnaire was used to record the sociodemographics of the participants |
| 2c | Abstract - Results What was found. Give the results for the main outcomes. | 1  Three patients, 186 males (62%) and 114 females (38%) participated. The prevalence of Helicobacter pylori infection was 67.7 percent (203/300). The prevalence of H pylori infection was significantly correlated with increasing age (p=0.012), sharing of a bed with siblings during childhood (p value=0.013) and the mode of sanitation methods (p= 0.023). There was no association found between H pylori infection and other risk factors such as; gender, level of education, employment status or number of rooms in a house. |
| 2d | Abstract - Conclusion - What have we learned and what does it mean. Where should future research go. | 1  H. pylori infection prevalence was significantly associated with increasing age, sharing of a bed with siblings during childhood and the mode of sanitation used. Clinicians and the public have to be aware of the important role of H pylori in upper gastrointestinal disease. Use of better sanitation methods, appropriate hygiene, avoidance of over-crowding amongst other measures should be encouraged as a means to reduce the acquisition and transmission of H pylori. |
| 3 | Explain the scientific background and rationale for the cohort study. What are objectives, research questions and the hypotheses. | 2  The specific objectives of this study were to:   1. Estimate the prevalence of Helicobacter pylori in asymptomatic individuals and,   2. determine the association of the Helicobacter pylori with potential risk factors such as age, gender and the sociodemographic status (level of education, number of rooms and family member living in/with, source of drinking water, sharing of a bed and animal ownership). |
| 4a | Registration and ethics State the research registry number in accordance with the declaration of Helsinki - "Every research study involving human subjects must be registered in a publicly accessible database before recruitment of the first subject" (this can be obtained from; ResearchRegistry.com or ClinicalTrials.gov or ISRCTN). Even retrospective studies should be registered prior to submission. | The study was registered Unique Identity Number (UIN) 2822 |
| 4b | Ethical Approval - State whether ethical approval was needed and if so, what the relevant judgement reference from the IRB or local ethics committee was? If ethical approval was not needed, state why. | 6  Permission to conduct the study was sought from the Joint Parirenyatwa Group of Hospitals and College of Health Sciences Research Ethics Committee, Ethics Committee at Harare Central Hospital and Clinical Directors of Parirenyatwa Group of Hospitals and Harare Central Hospitals. |
| 4c | Protocol - Was a research protocol developed apriori? Where can it be accessed. Was it published in a journal e.g. IJS Protocols, BMJ Open, etc, if so, provide the reference. | No research protocol was developed |
| 5a | Study design - State the research is a cohort study and whether prospective or retrospective in design, whether single or multi-centre. | 2  A cross-sectional study was done. Sample size calculated using the Dobson’s formula was 185. Between July 2014 to November 2014 four hundred and fifty patients were approached for the study and of these, three hundred were recruited.  Patients visiting the outpatient surgical clinics at two central, public and teaching hospitals for other complaints other than upper gastrointestinal symptoms were recruited in the study. Eligibility criteria was all individuals who did not have upper gastrointestinal symptoms presenting to the surgical outpatients’ clinics. Patients were excluded if; a) they had upper gastrointestinal tract symptoms such as epigastric pain, indigestion and nausea/vomiting, b) they had a history of peptic ulcer disease or any use of antacids regularly, c) they took antibiotics for the past 6 to 8 weeks, d) they were below the age of 1 year and e) if patients refused to be included in the study. The patients were recruited in the order of who was first in the outpatients’ queue.  Drops of whole blood were obtained by fingertip puncture from each patient. The commercial sandwich lateral flow chromatography kit (Onsite H. Pylori Combo Rapid Test, CTK Biotech) was then used to detect the presence of antibodies; immunoglobulin (Ig) G, Ig M or Ig A to confirm presence or absence of Helicobacter pylori. The Onsite H. pylori Combo Rapid Test has a relative sensitivity of 86.7 percent and relative specificity of 91 percent. A questionnaire was used to record the age, sex and socioeconomic status of the participants.  Most of the patients (n=80) excluded from the study had been on antibiotics. Ten patients who were eligible and had agreed did not turn up for the study after they had been attended to for their primary presenting complaints. All data from data collection sheet was entered into a computer using Epidemiological Information -programme software and was analyzed using Statistical Package for Social Scientist (SPSS) version 16. Descriptive statistics were used to report measures of central tendencies for quantitative variables. Student’s t-test for independent groups was used to test and also check relationships on continuous variables. Categorical variables were expressed as percentages and frequencies, and compared using the Chi-square analysis. Graphs were used to present categorical variables in pictorial view. Statistical analysis was carried out and P-value of 0.05 was considered significant at 95% confidence interval. |
| 5b | Setting - Describe the setting(s)and nature of the institution in which the patient was managed; academic, community or private practice setting? Location(s), and relevant dates, including periods of recruitment, exposure, follow-up, and data collection | 2  Patients visiting the outpatient surgical clinics at two central, public and teaching hospitals in Harare, Zimbabwe, for other complaints other than upper gastrointestinal symptoms were recruited in the study. Period was July 2014 to November 2014 |
| 5c | Cohort Groups - State the number of groups in the study. What interventions will each group receive? | It was a cross sectional study |
| 5d | Sub-group – Analysis. Any planned sub-group analyses are specified / Describe any methods used to examine subgroups and interactions. | No |
| 6a | Participants - State any eligibility (inclusion/exclusion) criteria and the sources and methods of selection of participants. Describe length and methods of follow-up. | 2  Eligibility criteria was all individuals who did not have upper gastrointestinal symptoms presenting to the surgical outpatients’ clinics. Patients were excluded if; a) they had upper gastrointestinal tract symptoms such as epigastric pain, indigestion and nausea/vomiting, b) they had a history of peptic ulcer disease or any use of antacids regularly, c) they took antibiotics for the past 6 to 8 weeks, d) they were below the age of 1 year and e) if patients refused to be included in the study. |
| 6b | Recruitment - State the methods of how patients or participants were recruited to each group, over what time periods. | 2  The patients were recruited in the order of who was first in the outpatients’ queue. |
| 6c | Sample size calculation Whether there was calculation of margin of error or a prior analysis to determine study population, or mention of how appropriate study sample was determined. | 2 and 3  Sample size calculation was done using the Dobson’s formula.  Calculation:  n = z^2^  x p (1-p)  d^2^  Description:  n= required sample size.  z= confidence level at 95% (standard value of 1.96)  p= estimated prevalence of H. pylori infection  d=margin of error at 5% (standard value of 0.05) |
| 7a | Pre-intervention considerations - e.g. Patient optimisation: measures taken prior to surgery or other intervention e.g. treating hypothermia/hypovolaemia/hypotension in burns patients, ICU care for sepsis, dealing with anticoagulation/other medications and so on. | None |
| 7b | Types of intervention(s) deployed - To include reasoning behind treatment offered (pharmacological, surgical, physiotherapy, psychological, preventive) and concurrent treatments (antibiotics, analgesia, anti-emetics, nil by mouth, VTE prophylaxis, etc). Medical devices should have manufacturer and model specifically mentioned. | None |
| 7c | Peri-intervention considerations - Administration of intervention (what, where, when and how was it done, including details for surgery; anaesthesia, patient position, use of tourniquet and other relevant equipment, preparation used, sutures, devices, surgical stage (1 or 2 stage, etc) and operative time. Pharmacological therapies should include formulation, dosage, strength, route and duration). Authors are encouraged to use figures, diagrams, photos, video and other multimedia to explain their intervention. | None |
| 7d | Who performed the procedure(s) - Operator experience for each group (position on the learning curve for the technique if established, specialisation and prior relevant training). | The author did the finger puncture and reading of the results |
| 7e | Quality control - What measures were taken to reduce inter or intra-operator variation. What measures were taken to ensure quality and consistency in the delivery of the intervention e.g. independent observers, lymph node counts, etc | One operator did the tests.  Indeterminate results were repeated. |
| 7f | Post-intervention considerations - e.g. post-operative instructions and place of care. Important follow-up measures - diagnostic and other test results. Future surveillance requirements - e.g. imaging surveillance of endovascular aneurysm repair (EVAR) or clinical exam/ultrasound of regional lymph nodes for skin cancer. | None |
| 8 | Outcomes - What primary and secondary (if any) outcomes will be assessed and how are they defined. Definitions should be clear and precise. Appropriate references to validation of outcome measures used should be provided if they exist. | None |
| 9 | Statistical methods - Clearly outlined statistical tests used to compare the outcomes between an intervention group and a comparison group, state whether pre-existing differences and known confounders were controlled.  The statistical package used should be mentioned. | It was a cross sectional study.  All data from data collection sheet was entered into a computer using Epidemiological Information -programme software and was analyzed using Statistical Package for Social Scientist (SPSS) version 16. Descriptive statistics were used to report measures of central tendencies for quantitative variables. Student’s t-test for independent groups was used to test and also check relationships on continuous variables. Categorical variables were expressed as percentages and frequencies, and compared using the Chi-square analysis. Graphs were used to present categorical variables in pictorial view. Statistical analysis was carried out and P-value of 0.05 was considered significant at 95% confidence interval. |
| 10a | Participants recruited with a flow diagram - Report numbers involved in each group and use a flow diagram to show recruitment, non-participation, cross-over, withdrawal from the study with reasons. | 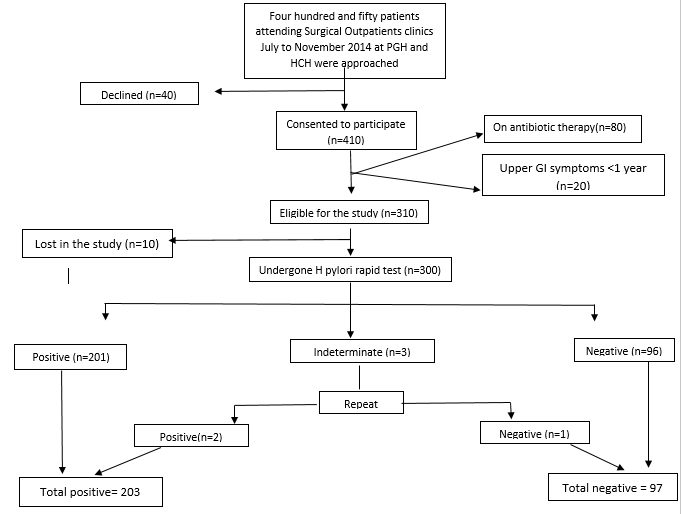 |
| 10b | Comparison between groups including a table - Provide a table comparing the demographic, clinical/prognostic features (co-morbidities, tumour staging, smoking status, etc) and relevant socioeconomic characteristics of each group and whether numerical differences are significant (using p-values and/or confidence intervals as appropriate). Were the groups matched and if so, how. | 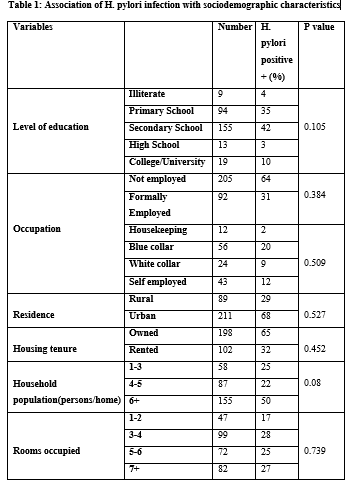 |
| 10c | Changes - Any changes in the interventions during the course of the study (how has it evolved, been altered or tinkered with, what learning occurred, etc) together with rationale and a diagram if appropriate. Degree of novelty for a surgical technique/device should be mentioned and a comment on learning curves should be made for new techniques/devices. | No |
| 11a | Outcomes and follow-up - Clinician assessed and patient-reported outcomes (when appropriate) should be stated for each group (size of effect with raw numbers and percentages) with inclusion of the time periods at which assessed. Relevant photographs/radiological images should be provided e.g. 12-month follow-up.Make it clear which confounders were adjusted for and which were not. | None |
| 11b | Intervention adherence/compliance and tolerability - How was this assessed. Describe loss to follow-up (express as a percentage and a fraction) or cross-over between group and any explanations for them. | None |
| 11c | Complications and adverse or unanticipated events - Described in detail and ideally categorised in accordance with the Clavien-Dindo Classification. How they were prevented, mitigated, diagnosed and managed. Blood loss, wound complications, re-exploration/revision surgery, 30-day post-op and long-term morbidity/mortality may need to be specified. | None |
| 12 | Summarise key results | Three patients, 186 males (62%) and 114 females (38%) participated. The prevalence of Helicobacter pylori infection was 67.7 percent (203/300). The prevalence of H pylori infection was significantly correlated with increasing age (p=0.012), sharing of a bed with siblings during childhood (p value=0.013) and the mode of sanitation methods (p= 0.023). There was no association found between H pylori infection and other risk factors such as; gender, level of education, employment status or number of rooms in a house. |
| 13 | Discussion of the relevance of the findings and rationale for conclusions - Relevant literature, implications for clinical practice guidelines, how have the indications for a new technique/device been refined and how do outcomes compare with established therapies and the prevailing gold standard should one exist and any relevant hypothesis generation. The rationale for any conclusions. | This study showed the prevalence of H pylori infection to be 67.7 %, a high prevalence which is similar to other studies conducted in developing countries. A prevalence of 86.8% was found among a South African population, 60.9% was found in Zambian population 90% in children and 85% in adults in Nigeria . Other studies showed the same trend of H pylori infection rates being high; 92% in Tanzania, 66.9% among Korean population , while a prevalence of 74.4%, 66% and 62% was found among Pakistan , Mexican and Chinese population respectively. Zimbabwe is still a developing country where poor methods of sanitation, overcrowding and low socioeconomic status contribute to the high prevalence of H. pylori as noted in this study. The study population consisted mostly of black Zimbabwean population |
| 14 | Strengths and limitations of the study | The derivation of the associated risk factors via a questionnaire which would be subject to bias is a limitation of the study .Two diagnostic tests would have increased the diagnostic power of the study. Use of ELISA would have been superior to finger prick test. Community based study as opposed to hospital based will in future be considered in future studies |
| 15 | State what needs to be done next, further research with what study design(s). | Community based study as opposed to hospital based will in future be considered in future studies |
| 16 | State the key conclusions from the study and key directions for future research | The high prevalence of H. pylori infection (67.7%) is comparable with data from other developing countries. H. pylori infection prevalence was significantly associated with increasing age, sharing of a bed with siblings during childhood and the mode of sanitation used. |
| 17a | State any conflicts of interest | None |
| 17b | State any sources of funding | None |
